# Insights for the substance use disorder care continuum: findings from a qualitative study with sexual and gender minority individuals who use methamphetamine

**DOI:** 10.1186/s13722-026-00710-1

**Published:** 2026-08-01

**Authors:** Conley Combs, Alexa B. D’Angelo, Kira Argenio, Drew A. Westmoreland, Raymond L. Moody, Adam W. Carrico, Christian Grov

**Affiliations:** 1 Department of Community Health and Social Sciences, Graduate School of Public Health and Health Policy CUNY, City University of New York (CUNY), 55 W 125 Street, 7th Floor, New York, NY 10027 USA; 2 CUNY Institute for Implementation Science in Population Health, New York, NY USA; 3 Department of Epidemiology, University of Florida, Gainesville, FL USA; 4 Department of Human Development and Family Sciences, University of Connecticut, Storrs, CT USA; 5 Department of Health Promotion and Disease Prevention, Florida International University, Miami, FL USA

**Keywords:** Methamphetamine, Sexual and gender minority, Substance use treatment, Recovery

## Abstract

**Background:**

Methamphetamine use continues to disproportionately affect sexual and gender minority (SGM) communities and is associated with physical, mental, and sexual health harms, including heightened HIV vulnerability. While prior epidemic waves have been well documented, the current resurgence (particularly among SGM individuals), remains underexplored and occurs within an evolving HIV prevention landscape. Persistent challenges within the substance use disorder care continuum (SUDCC), including stigma and limited treatment responsiveness, underscore the need for patient-centered clinical guidance. This study examines treatment navigation experiences of SGM individuals who use methamphetamine and their recommendations for improving their substance use care.

**Methods:**

Participants were recruited from a U.S. national HIV prevention cohort that oversampled for individuals reporting recent methamphetamine use. Twenty-nine participants completed semi-structured, in-depth interviews between December 2024 and January 2025. Interviews focused on healthcare experiences, treatment navigation, and participants’ recommendations for SUDCC stakeholders. Interviews were recorded and transcribed verbatim into written transcripts, which were analyzed using a codebook approach to thematic analysis.

**Results:**

Our findings were organized into four major themes: (1) *Variation in Motivation to Use Meth*, (2) *Need for Destigmatized Care*, (3) *Desire for SGM-Affirming Care*, and (4) *A Spectrum of Care Needs*. Participants reported a variety of motivations for using meth, including to enhance their sex lives, cognition, and emotion regulation—motivations that providers may benefit from understanding as they aim to address the overlapping physical and psychosocial health needs of patients who use methamphetamine. The importance of reducing stigma in clinical encounters was underscored frequently by participants, and represented a salient theme throughout interviews and across thematic categories. Participants described both experienced and anticipated stigma when underscoring the need for de-stigmatized substance use care, and illustrated how stigma hindered substance use care engagement and use of emergency services. Moreover, many reported a desire for community among other SGM individuals, particularly within group recovery contexts, but also elucidated the risks that accompanied drug network expansion within these treatment environments.

**Conclusion:**

This study advances the substance use and treatment literature by amplifying the voices of SGM individuals who use methamphetamine and providing guidance for providers who work directly with these populations.

## Introduction

Methamphetamine (meth) is a highly potent and addictive stimulant. Recreational meth is often distributed in crystal form and can be consumed via smoking, injecting, and snorting [1], or less commonly, taken orally and rectally [1, 2]. Individuals may use meth for its desirable effects, including euphoria, increased libido, energy, focus, executive functioning, and reduced inhibitions [3–5]. The extant research suggests that individuals may use meth recreationally to cope with past traumas [6], and to enhance social and sexual experiences [7, 8]. However, meth use is also associated with significant physical and mental health consequences, including increased risk for human immunodeficiency virus (HIV) transmission, tooth decay, cardiovascular events such as stroke or heart attack, and paranoia/psychotic episodes [1, 4, 9].

Sexual and gender minority (SGM) communities have been persistently, disproportionately affected by meth use since the early 2000s [1, 10–12]. In the early 2000s, federal regulation on over-the-counter ingredients used to make meth, and public health campaigns elucidating the dangers of meth contributed to a decrease in usage rates nationally [13–15]. During this previous era, meth was believed to be a drug affecting predominantly white, rural parts of the U.S [16, 17]. Meth use has since re-emerged in the queer party and hookup scene (e.g., sexualized drug use/chemsex) [11, 18]. Although emergent across the U.S., the current epidemic spike has seen increased usage in urban areas and among SGM individuals of color [11, 16, 19]. Namely, in this more recent rise in meth use, Black, Latino, and transgender groups within the SGM community are disproportionately affected by methamphetamine use disorder (MUD) [16, 19, 20]. Although, across SGM groups, including those that are assigned female at birth (e.g. bisexual women) there is increased concern for MUD [21]. Still, most of the literature focuses on assigned male at birth individuals (e.g. gay/bisexual men, transgender women, but less so non-binary individuals) [22]. Changing demographics of the groups most prominently impacted by MUD suggest that additional research and data are needed to understand the current iteration of the meth use epidemic, as shifting disparities present a substance use and sexual health concern.

Meth use is associated with heightened sexually transmitted infections (STI) and HIV incidence, which could be attributed to its disinhibiting, libido-enhancing effects [19, 23, 24]. Black, Latino, and transgender groups within the SGM community are disproportionately affected by HIV, and MUD may act as a significant contributor to widening racial/ethnic and gender disparities in HIV [16, 19, 20]. Indeed, one longitudinal cohort of SGM individuals vulnerable to HIV found that over one-third of new HIV diagnoses were among individuals who used methamphetamine persistently, with Black and Latino SGM individuals overrepresented among this group [25]. While the last meth use epidemic spike in the early 2000s in white, rural environments has been well researched, the current epidemic remains underexplored within the extant literature. Moreover, today’s meth use epidemic is implicitly affected by the updated HIV prevention landscape: the PrEP and undetectable=untransmittable (U = U) era. More specifically, while biomedical strategies for HIV prevention, such as PrEP, have emerged in the U.S., with expanding availability over the past decade, alongside improvements in HIV treatment facilitated by antiretroviral therapy (ART), uptake and adherence remains disproportionately lower among Black and Hispanic/Latino SGM adults [26–31]. Given the disparities observed in HIV incidence and its documented association with meth use [25, 32], as well as lower-than-needed uptake of PrEP medication and ART adherence [30, 31], critical efforts are needed to understand factors shaping meth use and related experiences among this community.

Beyond the need for further study of meth use within the evolving HIV prevention context, a greater understanding of stakeholders’ (e.g., service users or patients) experiences and recommendations for the Substance Use Disorder Care Continuum (SUDCC) is needed. Specifically, the SUDCC refers to the stages of prevention, identification, clinical treatment, and recovery support for all levels of substance use; from active addiction to substance initiation. It includes a broad range of services, care, and education: from prevention and early intervention through treatment (e.g. inpatient services), peer support (e.g. Crystal Meth Anonymous), to long-term recovery [33–36]. Although the continuum has sequential parts, movement through it may be iterative and non-linear. Moreover, the varied healthcare services that make up the continuum are subject to structural, payer and other access issues—varying by state and county-level SUDCC infrastructure and available services [37–40]. At the federal level, the Affordable Care Act requires coverage for behavioral health services including substance use treatment for most insurers, while the Mental Health Parity and Addiction Equity Act of 2008 dictates that coverage be “at parity” with medical and surgical services [41, 42]. However, in practice, many continue to face insurer and other service-level barriers to the SUDCC [38], particularly in rural areas, as well as Southeastern states and those that have not expanded Medicaid [40, 43, 44].

Additionally, existing evidence suggests that provider-level barriers to the SUDCC across levels of care and treatment may likewise undermine access to quality care for those who use meth [11, 45]. Indeed, one study reported that providers *perceived* that people who use meth posed a risk of assault (to them), had high recidivism into emergency departments and utilized more resources than their non-stimulant-using counterparts [46]. Furthermore, providers have reported a need for training regarding sexualized drug use, enhanced referral pathways, and more pharmacological treatment options for MUD [47, 48]. Community members and service users have similarly reported a desire for longer-term care and insufficient linkage to care [45, 49, 50]. Prior qualitative research has extensively documented experiences of stigma reported both by SGM individuals seeking care, including internalized and enacted or social stigma [5, 7, 45, 50–53], *and* by healthcare providers [54]. To illustrate this, a recent qualitative study found that while reducing meth use in sexual contexts was a prominent goal reported among SGM individuals engaging in counseling or therapy, anticipated provider stigma often prevented disclosure of sexualized use [55]. More broadly, research suggests that experiences of stigma led individuals who used meth to feel like unworthy patients and cultivated internalized and anticipatory stigma, impeding access to providers, healthcare utilization and treatment engagement [51, 53, 54].

Taken together, there is a need to explore the role SUDCC services play in the relationship between meth use and sub-optimal health for SGM people, particularly in the modern era of the HIV prevention and treatment landscape. Further study is needed to identify practical clinical directions to address high rates of meth use. In other words, what do providers need to know to refine and reduce stress on the substance use disorder care continuum? This study seeks to fill this gap in research by presenting the experiences of a sample of SGM individuals, namely sexual minority individuals who use meth, and their recommendations for the SUDCC.

## Methods

### Participants and recruitment

Participants in this study were recruited from its parent study, the American Transformative HIV Study (AMETHST) which is an ongoing HIV prevention cohort study (2022–2027) with ~ 5,000 participants. AMETHST is a U.S. national study which oversampled men of color and men who reported using methamphetamine in the last three months, aged 16–49 years old [56]. Transgender men, transgender women, and nonbinary people who have sex with men were also represented in the AMETHST cohort but were not intentionally oversampled for this qualitative study. Eligibility criteria included reporting meth use in the past month. Participants were then sampled by stratified racial/ethnic categories of Black, Latinx, and White/all other races. Participants were provided a $40 gift card for completing the interview. Interviews began in November 2024 and concluded in January 2025.

### Measures and procedure

Between December 2024 and January 2025, 29 individuals participated in in-depth interviews via video-conferencing software. Mean interview time was 54.9 min, ranging from 29.4 to 82.2 min. The first author took extensive field notes that documented meth use patterns (e.g., daily, biweekly), initial ideas, methodological considerations, and reflexive personal notes. Recruitment was concluded at 29 participants as field notes showed that data collection was no longer yielding new analytic insights. Interviews were semi-structured following an interview guide, which explored experiences both past and present with meth, sex, other drugs, and navigating substance use treatment. The present study utilizes data primarily from the portion of interviews that broadly explored treatment navigation experiences and recommendations for healthcare providers and other people who use meth. The concluding question asked: “What are your recommendations for ways to support LGBT people who use meth?” Interviews were recorded, transcribed verbatim, and de-identified. Pseudonyms are used to protect participants’ privacy. Written informed consent was obtained during the email exchange as a part of the recruitment process. Participants were asked for an additional level of oral consent during the interview to ensure comfort with being recorded and allow time for questions. The Institutional Review Board of the City University of New York approved study procedures.

### Analysis

A codebook approach to thematic analysis was employed, while drawing from Braun and Clarke’s six-phases of reflexive thematic analysis [57–59]. A preliminary topical codebook was developed using our field notes, where preliminary codes and early topics were outlined through a reflexive and iterative process of engagement with case-by-case notes documented in Excel. For this analysis, we chose to focus on participants’ recommendations for medical providers and stakeholders, as this topic represented an important, actionable area of our data with rich narrative data across interviews. Upon selecting this topic, we expanded our analysis through a systematic reexamination of all transcripts.

The following analytic steps outline the processes taken by the first author (CC). First, field notes were open coded to create a preliminary codebook that was used to categorically organize salient topics recorded across interviews. After choosing the central focus for this analysis from our preliminary codebook of topics, the first-author began by open coding the transcripts, progressing to increasingly focused coding through an iterative process of refinement and reorganization of codes. In this coding process, new codes were developed and existing descriptive codes evolved to become more interpretive. The final codebook included 7 parent codes and 20 child codes, which are reported in our Results within four broad themes. Peer debriefing sessions with the second author (AD) occurred between field note writing, preliminary codebook development, during the iterative process of refining and naming themes, and throughout the manuscript writing process. Demographics reported for this study were taken from participants’ 12-month assessment from the broader parent study.

## Results

The average age of participants was 37.9 years old with a standard deviation of 6.05 years. Participants were split into three categories: Black (*n* = 10; 34.5%,), Latino (*n* = 10; 34.5%), and “all other races” (*n* = 9; 30.9%), which included a majority White individuals, one Asian individual and a multiracial person (See Table 1). Participants were mostly gay (*n* = 24; 82.8%) and cisgender (*n* = 25; 86.2%), although other queer sexualities and minority gender identities were included, for example, two non-binary and one transgender participant. 13/29 participants did not have a primary care provider (*n* = 13; 44.8%), and over half had an income less than $30,000 a year (*n* = 15; 51.7%). Over half (*n* = 15; 51.7%) reported using meth 21 days or more over the three months prior to their 12-month assessment (the most recent complete dataset at the time), and 13 (44.8%) participants reported having injected meth in the past year. Participants reported engaging with the SUDCC at a range of stages, including via harm reduction services (e.g. syringe access services), primary care, mental health counseling and therapy, psychiatric care, emergency medical care, substance use treatment (e.g. inpatient/residential treatment, intensive outpatient programs, and outpatient treatment), case management and social services, peer support groups (e.g., AA, NA, and Crystal Meth Anonymous), and recovery coaching or peer navigation. Our findings were organized into four major themes: (1)* Variation in Motivation to Use Meth, *(2)* Need for Destigmatized Care, *(3)* Desire for SGM-Affirming Care, and *(4)* A Spectrum of Treatment Preferences*. 

**Table 1:** Demographic characteristics of participants, *N* = 29

Characteristics	M ± SD or *n* (%)	
**Age**	37.9 ± 6.05	
**Race/Ethnicity**		
White	7 (24.1%)	
Asian	1 (3.4%)	
Black	10 (34.5%)	
Latinx/Latino	10 (34.5%)	
Multiracial/Other	1 (3.4%)	
**Sexual Orientation**		
Gay	24 (82.8%)	
Bisexual	2 (6.9%)	
Pansexual	1 (3.4%)	
Other	2 (6.8%)	
**Gender**		
Cisgender man	25 (86.2%)	
Transgender woman	1 (3.4%)	
Non-binary, genderqueer, genderfluid	2 (6.9%)	
**Region**		
Northeast	1 (3.4%)	
Midwest	4 (13.8%)	
South	17 (58.6%)	
West	7 (24.1%)	
**Have a primary care provider?**		
Yes	16 (55.2%)	
No	13 (44.8%)	
**Employment**		
Full time	11 (37.9%)	
Part time	5 (17.2%)	
Part-time work – Full-time student	1 (3.4%)	
Permanent or temporary disabled and not working	2 (6.9%)	
Permanent or temporary disabled but working “off the books” or “under the table”	2 (6.9%)	
Unemployed – Student	2 (6.9%)	
Unemployment – other	6 (20.7%)	
**Income**		
Less than $10,000	8 (27.6%)	
$10,000 to $29,999	7 (24.1%)	
$30,000 to $39,999	5 (17.2%)	
$40,000 to $49,999	3 (10.3%)	
$50,000 or more	6 (20.7%)	
**Injected meth in past year?**		
Yes	13 (44.8%)	
No	16 (55.2%)	
**Number of days using meth in past 3 months (collected at baseline)**		
I have not used meth in past 3 months	3 (10.3%)	
1–10 days	8 (27.6%)	
11–20 days	3 (10.3%)	
21 + days	15 (51.7%)	

## Variation in motivation to use meth

Participants in this study reported three primary underlying motivations to use meth: executive functioning/stimulation, sexual enhancement, and emotional well-being. Participants described using meth for focus, executive functioning, and stimulation (energizing effects). Several participants reported replacing Adderall with meth (or vice versa) to self-medicate for ADHD. In some cases, participants used meth to enhance their productivity and endure unsustainable workloads (e.g., being a full-time student and working a full-time night job, working two full time jobs). Across this motivation domain participants likened their meth use to a “cup of coffee in the morning,” (Henry, age 42, White, Cisgender Man). This characterization speaks to how participants view their own meth use ritual as less severe than generally portrayed.

Participants used meth to improve their sex lives, reporting increased libido, confidence, and disinhibition. However, meth and sex became increasingly ritualized together. Nearly all (28/29) participants reported how meth was deeply intertwined with their sexual lives—with most reporting disinterest in sex without meth, saying they only had sex while high and/or exclusively with other SGM individuals who ‘parTy.’ This type of motivation was particularly potent for participants whose connection between sex and meth use felt inextricably linked and difficult or impossible to separate.

Lastly, and reported amongst all participants (29/29), meth was used to enhance individuals’ emotional well-being and/or regulate their emotions, especially in the case of depression, PTSD/acute trauma, and anxiety (sexual anxiety included). Participants illustrated how complex traumatic experiences motivated their use of meth, as they turned to meth to help manage their emotions in lieu of healthcare intervention:

After the first few times I’ve used, I noticed something that turned off and it was like I could breathe for the first time in my life. I didn’t have the voices in my head… and yes, the raunchy sex and multiple partners and all the other fun stuff, I mean that was fun too, but it was just that feeling of freedom and being able to breathe that appealed to me, – Scott, age 42, White, Cisgender Man

The domain of emotion regulation was robustly self-reported across interviews, with participants reporting using meth to manage a breadth of emotional experiences, particularly those that were distressing. Some participants characterized their self-medication like a cigarette after a hard day of work, ostensibly using it as a strategy to de-stress.

Given that many participants are motivated to use meth to enhance their cognition, sexual lives, and for emotion regulation, it is essential that healthcare providers understand these underlying causes, as patients may require support to address sexual, psychological, and emotional challenges as part of their broader substance use care. For instance, addressing access to psychiatric care for ADHD, specialized sex therapy, and other mental health services may benefit those who are actively using methamphetamine.

### Need for destigmatized care

The subject of stigma arose across themes, including *A Spectrum of Treatment Preferences* and *Desire for SGM-Affirming Care*, but was raised with such frequency and saliency as to constitute its own distinct theme. Illustrating the varied experiences of stigma, participants described being called “disgusting” online to being denied pain medication by an emergency room doctor after being documented as a meth ‘addict’ (Kaley, age 47, White, Transgender Woman). Beyond concerns about stigma and homophobia, participants reported that fear of legal repercussions hindered utilization of healthcare and emergency services—these distinct but overlapping challenges undermined navigation of the SUDCC. Even in emergencies involving unconsciousness and overdose, “people don’t like to call the ambulance or the police when stuff like that happens, because it’s scary. Or you may go to jail,” (Diego, age 33, Latino, Cisgender Man).This stigma-based fear made some participants apprehensive to enter the continuum at all.

Participants expressed a variety of concerns when utilizing healthcare services, most commonly, discomfort communicating with providers and challenges finding providers who met their needs (e.g., harm reduction approach to drug use, SGM-affirming). Some participants were fortunate to have compassionate primary care physicians who monitored for potential health consequences of meth use without moralizing their patients. However, many encountered judgment:

I talk to the doctors, it’s like, I want to tell them the truth, but I’ve done that before… and then they just judge me, and all of a sudden they treat you bad… Like you’re just a piece of shit, and they don’t really care about you, – Kaley, age 47, White, Transgender Woman

This participant’s desire to be honest about their drug use illustrates their intent to take care of themself, but it was curbed by anticipated provider stigma. One participant emphasized the need to decouple stigma from the medical context, saying:

To providers, I would say check the judgment at the door. I’ve never had really judgmental healthcare providers, but I have friends who are doctors… some of them can be a little nose in the air, which doesn’t make any sense to me in the field that you’ve chosen and the oath that you’ve taken. I’d say don’t forget your oath – Jayden, age 39, Black, Cisgender Man

As articulated by this participant, stigma is counterproductive to the aims of healthcare and the mission of providers.

Provider stigma is only part of a greater societal stigma, which participants felt pervasively. Furthermore, participants reported feelings of social exclusion, which contributed to the belief that the “parTy and play” (PnP) community is perceived as a deviant, underground community isolated from society. The SUDCC can exacerbate feelings of social isolation; one participant critiqued in-patient treatment programs saying, “I don’t think that I have to be removed from society in order to get rid of my habit,” (Raúl, age 43, Latino, Non-binary). Meanwhile, another participant spoke of experiencing broader mainstream stigma:

That’s what I feel is needed in this community. Just more guidance, more help. We don’t get a lot of that, not from outsiders. We may get that support maybe from the circle [the queer and PnP community], but not from outside of the circle, for sure, – Isaiah, age 37, Black, Cisgender Man

Another participant reported experiencing support from his stepmother–a heterosexual individual who does not use meth (i.e., an ‘outsider’)–but who nonetheless aided in his recovery. Having her nonjudgmental support was essential to his recovery, reiterating the harm of ostracization and necessity of acceptance from broader society.

## Desire for SGM-affirming care

Participants’ experiences of judgment and discomfort with medical providers and broader society bolstered the need for SGM-affirming care. Participants described feeling like an outsider within heteronormative society and struggled “to feel accepted completely” (Ale, age 36, Latino, Cisgender Man) by the SGM community due to their meth use. Many expressed a desire for SGM community within care settings where they could “cope together” (Diego, age 33, Latino, Cisgender Man). Participants found Narcotics Anonymous particularly affirming, even outside of better LGBTQ-resourced major cities. One participant articulated how the normalization of their experience within a queer group therapy context was a gateway to recovery, saying:

One of the things that brought me so much comfort through the process of recovery was that my story was not unique… it’s not until you’re sitting in a room with other addicts and they’re telling your story exactly the stuff that you do. And for some reason that brought me comfort because I felt like, okay, I’m not crazy. I’m not something that’s totally bizarre … other people do the stuff that I’m doing. – Henry, age 42, White, Cisgender Man

This participant underscored how having a network of recovering SGM friends supported their own recovery. Altogether, participants reported that SGM-affirming services improved recovery outcomes.

Likewise, participants described how discomfort stalled recovery. One participant reported the long and unfruitful search for an affirming mental healthcare provider:

After the difficulty of finding anyone you can be comfortable enough to talk to about something like this and who you don’t feel is judging you, [they were fired]. – Ace, age 28, Black, Cisgender Man

At the time of their interview, the participant did not have a new provider. Effectively, the lack of accessible, SGM-affirming providers using a harm reduction approach presented a continuity of care issue. Another participant who sought faith-based substance use programming encountered homophobic clergy in their search, which led to their relapse (Ale, age 36, Latino, Cisgender Man). Overall, the difficulty of finding an SGM safe space within the SUDCC appeared to impact recovery outcomes.

## A spectrum of treatment preferences

Participants navigated the stages of the SUDCC linearly, non-linearly, and iteratively, to varying degrees of satisfaction, while highlighting some risks. For example, some participants found that the social dynamics of 12-step programs (e.g., sponsorship, meeting secretary) were distressing and undermined their recovery. One participant encountered interpersonal conflict or ‘drama’ within their Alcoholics Anonymous (AA) program (Jackson, age 41, White, Cisgender Man). They preferred Narcotics Anonymous (NA) over AA, where they felt less judgment and more community support. Another participant found their leadership position overwhelming: “It’s a lot of pressure because so many people,” … “[they] expect so much from you,” (Isaiah, age 37, Black, Cisgender Man), which factored into their relapse. Relatedly, one participant mentioned that their experience in 12-step programs suffered from a lack of personal availability within the community, stating that *if* “there was somebody there that you would call, and they would actually pick up the phone. It would make a world of difference,” – John-Henry, age 40, Latino, Cisgender Man.

For some participants, the group recovery environment may exacerbate the risk of relapse. Several participants found the group environment where meth was over-discussed unhelpful, as it only increased cravings and temptation. For some, these environments expanded their social networks for using meth.

Sitting in a room with a group of tweakers and ex-tweakers, as I put it, was not conducive to my sobriety. It made me almost want to go out and use after going to one of those meetings. – Connor, age 40, White, Cisgender Man

Likewise, one participant reported that after seven in-patient treatment episodes, they subsequently relapsed each time with people they met in treatment (Kevin, age 33, Black, Cisgender Man). Other participants problematized Crystal Meth Anonymous (CMA) specifically, saying:

I’m in NA, but I’ve been to Meth-Anonymous, and I didn’t like it because there was people that are using and people that aren’t using in the same program, and that won’t work… So yeah, Narcotics Anonymous, it’s like AA so it holds a little bit more of a standard on you. They hold a little more accountability to you. And you have to recover and use accountability. Without it, it just doesn’t work. – Raúl, age 43, Latino, Non-binary

This participant illustrated how the social network established in a group recovery environment has an important impact on recovery and relapse, while underscoring how some programs may be more successful than others for those who use meth.

Many participants reported how recovery requires changing their social network and/or physical environment (e.g., switching friend groups, moving states). One participant described disconnecting from their friends who used meth:

I’m like the one sober person sitting there on the couch and just watching the circus of people coming and going and listening to the bullshit. And there are little dumb dramas they have with each other, and it’s kind of surreal, but I didn’t feel like I’d fit in anywhere else. Those were my people. I don’t have other people. So, I think that was one of the hardest parts about it, – Jackson, age 41, White, Cisgender Man

Therefore, not having a support system of sober friends appeared to complicate recovery. Amongst participants overall, leaving their meth network was necessary, but for others, developing a social network of those in recovery also posed a risk.

## Discussion

This study adds to the body of literature on the methamphetamine epidemic in the U.S. by characterizing meth use motivations and the SUDCC experiences and preferences of SGM individuals who use meth. Our findings echo past studies and address gaps in knowledge with regard to providing care for those who use meth. Collectively, our results suggest that SGM individuals have varied motivations for using meth and would benefit from an array of choices when considering treatment options. Healthcare providers, particularly those who are touch points in the SUDCC, should be nimble and well-trained to tailor treatment plans to patients’ use patterns and treatment preferences, while employing de-stigmatizing and LGBTQ+ affirming communications. Below, we outline our four primary findings, situating them within the extant literature, and exploring how they may be used to inform clinical encounters.

First, understanding the underlying motivation to use meth can help providers guide patients appropriately along the care continuum. Extant research has reported a growing understanding of the nuanced reasons why SGM individuals use meth. For example, studies have reported that people use meth to relieve themselves from experiences of childhood abuse and insidious trauma [6], to enhance sexual experiences [3, 60–62], and non-sexually, including in the SGM nightlife scene, sometimes for social motivations [18, 62]. Thus, the literature supports our findings that people use meth for their emotional well-being and sexual lives. Additionally, our study illuminates that SGM individuals also use meth as a stimulant to enhance productivity and executive functioning; sometimes even to self-medicate for ADHD. Thus, providers should be prepared to link patients to ADHD care to meet the mental health needs of patients who use meth. Tailoring treatment to the underlying reasons people use is a more compassionate alternative to stigmatizing abstinence-based drug treatment approaches.

Second, our findings support that stigma from providers acts as a robust barrier to treatment, with the potential to disrupt continuity of care and entry into the SUDCC. Meth-related stigma is not merely a social-moral issue; it has tangible health consequences [51]. For instance, some participants were so fatigued by stigmatizing interactions and the challenge of trying to find a provider they were comfortable with (i.e. queer affirming with a harm-reduction approach to drugs), that they fell out of the SUDCC entirely. Those who remained in the SUDCC still encountered medical discrimination because of this stigma. Therefore, good provider-patient relationships are very vulnerable to stigma but critical for the continuity of care for this group. Moreover, recognizing the enacted stigma that SGM communities have encountered within the care context may help inform the implementation of trauma-informed delivery, aligning with recognized clinical care guidelines, including those well established in non-U.S. contexts [63].

Moreover, emergency departments (ED) and emergency medicine services (EMS) are an opportune area for refinement of the SUDCC and intervention for SGM individuals who use meth. ED/EMS are a primary entry point into the SUDCC [50–52, 54]. Stigma has been reported specifically in ED/EMS by some participants in the present study and the extant literature [50–52, 54]. Stigma in ED/EMS, coupled with some participants’ reluctance to utilize emergency services out of fear of legal consequences, can help explain existing findings that report less than a quarter of participants exhibiting meth overdose symptoms utilize ED/EMS [64]. People who use methamphetamine that end up in emergency services would benefit from provider interactions that emphasize compassion, as well as education on their state-specific rights and legal protections to prevent overdose deaths.

Altogether, stigma is ineffective in deterring meth use and instead generates medical mistrust, fatigue in navigating services, and deters life-saving care. Providers should work to de-stigmatize meth use through practice. For instance, encourage open dialogue with patients about their drug use, forgoing moralizing language, which undermines the provider-patient relationships and comfort of SGM individuals who use meth. Interestingly, participants in this study never reported that stigma kept them from using; instead, it may have fueled their use. The social taboo of using meth was eroticized as a form of sexual liberation and affected care outcomes. Indeed, extant evidence suggests that many who use meth eventually “age out” of use, as individuals encounter cues in life that trigger cessation [65]. Thus, prioritizing de-stigmatizing, harm reduction strategies that prevent overdose may allow for individuals to reach a stage in life where they are ready to reduce or cease use of meth [66].

Third, non-SGM-affirming care impacted care outcomes, and participants sought SGM-affirming care pathways. Existing literature already supports that identity-based stigmas against SGM individuals negatively impact health outcomes [67], and underscores the value of SGM affirmation within the context of substance use interventions [49, 67–71]. Perhaps this is why a majority of experimental SGM-tailored chemsex interventions, which have been examined in the literature, are rated with high acceptability and satisfaction by participants; yet, these are not commonplace within the SUDCC [49]. Studies that examine non-SGM tailored entry points into the SUDCC, such as inpatient hospitals and emergency departments, reported low patient satisfaction [45, 50, 70]. Some participants’ experiences with SGM affirmation in group recovery environments available in the SUDCC provide insight into the next primary takeaway—the potential of social network expansion.

SGM-affirming care is essential to community building within the SUDCC, which can be a core mechanism of recovery for SGM individuals. When the SUDCC is a safe space for SGM individuals, it allows them to build new relationships in healthier recovery spaces as opposed to seeking community in the “parTy” scene. Additionally, efforts to create SGM community within recovery approaches should be inclusive of assigned female at birth SGM people who also experience higher rates of meth usage yet are underrepresented in the literature. Our data support that participants found belonging in 12-step programs like Narcotics Anonymous because it was a space where they could “cope together.” Given that participants emphasized the necessity of leaving their social network of peers who actively used, and some 12-step programs were reported as valuable social experiences, the construction of healthier social networks is a necessary function of the SUDCC. Meth networks can be replaced with recovery networks as a form of targeted SGM community development, which is reported by our participants and extant literature as a desired path to recovery [49, 68, 70, 72].

Last—in regard to theme four—there is great variation in the SUDCC pathways that suit the individual needs of patients, and thus, improvements to the SUDCC should focus on taking a patient-centered approach to care. Our findings elucidated a spectrum of care needs, from communal to individualized, and highlighted risks intrinsic to recovery models, which represented a particularly salient issue among some participants. Although we have just underscored the value of SGM community building in group-based treatment, we must also acknowledge that participants connected experiences of relapse to group treatment. Some participants described group discussions of meth (“war stories”) as potentially triggering the desire to use meth again. Moreover, another challenge that arose in our data—not yet sufficiently documented in the meth literature—is that some group therapy environments in the SUDCC run the risk of expanding active meth using social networks [73–75]. Given that some participants reported that 12-step programs (especially CMA), rehab, or residential programs connected them to the very people they relapsed with, further research and intervention are needed to address this barrier to recovery. Such realities have been underexplored in the literature, and by extension, effective intervention for this issue currently eludes us. Ultimately, our data highlighted participants who both repudiated and celebrated group recovery environments for these very reasons.

We have argued simultaneously that SGM individuals who use meth benefit from leaving their social network *and* need an SGM-affirming community at the same time. This seeming contradiction reveals an important dichotomy: some participants found the group recovery context to be fundamentally unhelpful, while others found it essential to recovery. A spectrum of care needs from individual to communal should be promoted to patients. Providers should be knowledgeable and transparent about the benefits and risks of group recovery services when recommending/linking patients to group services, and provide patients with enough agency to choose the services that best fit their needs. Recovery pathways that replace old social networks with sober, SGM communities *or* separate patients from active meth using networks are both valid.

Finally, although group-based recovery models remain foundational within the substance use disorder care continuum [76, 77], our findings suggest that some 12-step groups may be operating under structural capacity constraints—with one participant in leadership reporting being overextended by 12-step responsibilities, and another requiring greater support from sponsors. Given that most 12-step programs are autonomous and primarily supported through member contributions, limited resources may restrict the availability of sponsorship for individuals early in recovery, while simultaneously increasing service demands on members with more established sobriety. This dynamic may elevate burnout risk among sponsors and group leaders, particularly in smaller or more specialized fellowships. These challenges may be especially pronounced within CMA groups serving SGM populations, where membership size is often smaller and members may have distinct recovery and psychosocial support needs compared to larger fellowships such as AA or NA. Rather than reflecting shortcomings of 12-step programs themselves, these findings underscore the importance of sustainable funding mechanisms, healthcare partnerships, and community investment to support peer-led recovery infrastructures without placing additional burden on volunteer members.

### Limitations

Qualitative study designs have a unique ability to generate rich data, context, and provide unique insights into the experiences of individuals; however, these designs are not without their limitations. For example, while not a goal of this study, we cannot say to what extent these findings lend themselves to demographic transferability. Although this study included one transgender woman and two nonbinary people, it cannot speak reliably to these groups’ experiences. Although our study oversampled persons of color (i.e., two-thirds of the sample), it only included one Asian individual. The range of Asian identities could have distinct cultural experiences of meth use and recovery in the PnP community. Future research studies should consider oversampling gender diverse participants and all people of color, which may reveal unique implications for stigma, directions for intervention, and motivations for using meth. Relatedly, while the impetus for this study emerged in consideration of meth use within the HIV prevention landscape and persistent racial/ethnic disparities in care access, the current study was not designed to elicit participants’ experiences related to HIV prevention or treatment engagement given their extensive documentation in extant literature. However, future research should consider the implications of our findings relating to meth use motivations and care needs within the context of the evolving HIV care environment. Further, this study aims to guide healthcare practice yet did not include interviews with providers. By focusing on patients, our study could be missing practical provider perspectives for implementation. While our use of reflexive thematic analysis allowed us to describe nuanced care preferences, it was not suitable or intended to comprehensively inventory the breadth of treatment modalities utilized by the sample. Thus, additional quantitative research should assess the frequency and prevalence of specific treatment modalities and preferences among this population.

## Conclusion

In conclusion, the supporting literature suggests that contemporary care methods for SGM individuals who use meth are inadequate. Our study suggests that stigma undermines SGM individuals’ experiences with the SUDCC, resolvable through community building via SGM-affirming care. However, in the process of community building, connecting with new individuals who maintain active meth use while attempting recovery may pose a risk— and is currently an understudied challenge with implications for an emergent dichotomy of care needs. Additionally, our data suggest that understanding SGM individuals’ motivations to use meth can offer critical insights into tailoring care for this population. Informed by these data, we propose four primary recommendations for providers that can improve SUDCC’s ability to combat high rates of meth use. This study aimed to support providers who treat this population with the hope of strengthening care practices, destigmatizing meth use, and supporting effective clinical practices that meet the diverse needs of SGM individuals who use meth. Our study advances this literature, raising more alarms for the federal declaration of the meth epidemic as a national public health emergency, as was done for opioids. National intervention could fund de-stigmatizing campaigns, commit resources, offer training in SGM-affirming care, and support research that further explores interventions tailored to the spectrum of care needs and use motivation.

## Data Availability

Data from this study are available upon reasonable request.
